# The intergenerational multiple deficit model and the case of dyslexia

**DOI:** 10.3389/fnhum.2014.00346

**Published:** 2014-06-02

**Authors:** Elsje van Bergen, Aryan van der Leij, Peter F. de Jong

**Affiliations:** ^1^Department of Experimental Psychology, University of OxfordOxford, UK; ^2^Research Institute of Child Development and Education, University of AmsterdamAmsterdam, Netherlands

**Keywords:** intergenerational multiple deficit model, generalist genes hypothesis, dyslexia, comorbidity, family risk, developmental disorders, intergenerational transmission

## Abstract

Which children go on to develop dyslexia? Since dyslexia has a multifactorial etiology, this question can be restated as: what are the factors that put children at high risk for developing dyslexia? It is argued that a useful theoretical framework to address this question is Pennington’s (2006) multiple deficit model (MDM). This model replaces models that attribute dyslexia to a single underlying cause. Subsequently, the generalist genes hypothesis for learning (dis)abilities (Plomin and Kovas, 2005) is described and integrated with the MDM. Next, findings are presented from a longitudinal study with children at family risk for dyslexia. Such studies can contribute to testing and specifying the MDM. In this study, risk factors at both the child and family level were investigated. This led to the proposed intergenerational MDM, in which both parents confer liability via intertwined genetic and environmental pathways. Future scientific directions are discussed to investigate parent-offspring resemblance and transmission patterns, which will shed new light on disorder etiology.

## PROBLEMS FOR SINGLE DEFICIT ACCOUNTS OF DYSLEXIA

Research into dyslexia has been dominated by the quest for the Holy Grail: the single cognitive deficit that is necessary and sufficient to cause all behavioural characteristics of the disorder. The dominant hypothesis of this kind has been the phonological-deficit hypothesis (e.g., Wagner, 1986; Snowling, 1995). However, a single cognitive deficit model of dyslexia, as single deficit models of developmental disorders in general (see Pennington, 2006, for a comprehensive overview), has a number of shortcomings. First, there is no single cognitive deficit found that can explain all behavioural symptoms of all cases with dyslexia (e.g., Ramus and Ahissar, 2012). For example, not all individuals with dyslexia show a phonological deficit (e.g., Valdois et al., 2011; Pennington et al., 2012). Conversely, not all individuals with a phonological deficit have dyslexia (e.g., Snowling, 2008; van der Leij et al., under review). This questions a one-to-one mapping and points to the possibility that various constellations of underlying cognitive deficits can lead to the behavioural symptoms of dyslexia.

In addition, a single deficit model cannot readily explain the phenomenon of comorbidity. For instance, dyslexia co-occurs more often than expected by chance with other developmental disorders, including dyscalculia, specific language impairment (SLI), speech-sound disorder, and attention-deficit/hyperactivity disorder (ADHD). To illustrate this point, suppose disorder A and B each have a prevalence of 5% in the general population. If disorder A and B were independent, then 5% of the cases with A would also have B. However, comorbidity rates for developmental disorders are commonly in the order of 30%, for example between dyslexia and speech-sound disorder or dyslexia and ADHD (Pennington, 2006). The huge discrepancy between these figures (5 vs. 30%) implies that developmental disorders are not independent.

The single deficit model requires for each comorbidity (pair of disorders) a distinct account. Pennington (2006) discusses as an example the comorbidity between dyslexia and speech-sound disorder. Speech-sound disorder is defined by difficulties in the development of spoken language, especially problems with the intelligible production of speech sounds. Approximately 30% of children with early language or speech problems go on to develop dyslexia. A parsimonious single deficit model to explain this comorbidity is the severity hypothesis. The severity hypothesis states that speech-sound disorder and dyslexia have the same underlying phonological deficit, with speech-sound disorder being an earlier developmental manifestation of this deficit than dyslexia. Comorbid cases will have the most severe phonological deficit. If the phonological deficit is less severe, speech-sound disorder will not reach clinical boundaries but dyslexia will. To account for cases with early speech-sound disorder but without later dyslexia, the model must pose a subtype of speech-sound disorders that is caused by a phonological deficit distinct from the phonological deficit as seen in cases with dyslexia. Alternatively, the phonological deficit in such cases must be resolved by the time they come to the task of learning to read. However, Snowling et al. (2000) followed a group of former language-impaired children into adolescence. Those with early speech-sound disorder (isolated phonological impairments at 4 years of age) had normal reading skills at age 15, but continued to show phonological deficits. Similar results were obtained by Peterson et al. (2009). In their study many children with early speech-sound disorder went on to learn to read normally despite a lasting phonological deficit. Thus, in both studies the children with early speech-sound disorder had a phonological deficit similar to children with dyslexia. This conclusion is inconsistent with the single cognitive deficit severity hypothesis.

While research at the cognitive level of explanation was still searching for a single deficit, studies at the genetic level converged on the conclusion that the aetiology of dyslexia, as of other developmental disorders, is genetically complex (Pennington, 2006). So instead of a single gene determining dyslexia, many genes act probabilistically (i.e., polygenicity), each having only a small contributory effect to the etiology of dyslexia (Bishop, 2009). Moreover, behavioural genetic studies have shown for certain developmental disorders that the relation between two traits (like reading ability and inattention) is larger in monozygotic (MZ) twin pairs than in dizygotic (DZ) twin pairs (Willcutt et al., 2000). Such a bivariate heritability supports genetic overlap between the conditions, in this example between dyslexia and ADHD. The partly shared etiology of dyslexia and ADHD does not yet rule out the possibility of a distinct single cognitive deficit for each disorder. However, studies have demonstrated that a processing speed impairment is not only a characteristic of dyslexia, but also of ADHD (e.g., Willcutt et al., 2005), suggesting that processing speed is a shared cognitive risk factor (McGrath et al., 2011). The accumulating evidence for etiological and cognitive overlap between dyslexia and ADHD speaks against a single deficit model for explaining their frequent co-occurrence. Also for other dyslexia comorbidities, shared cognitive deficits are found, for example a phonological deficit in SLI (e.g., Bishop et al., 2009) and a processing-speed deficit in dyscalculia (e.g., van der Sluis et al., 2004).

## THE MULTIPLE DEFICIT MODEL

**FIGURE 1:**
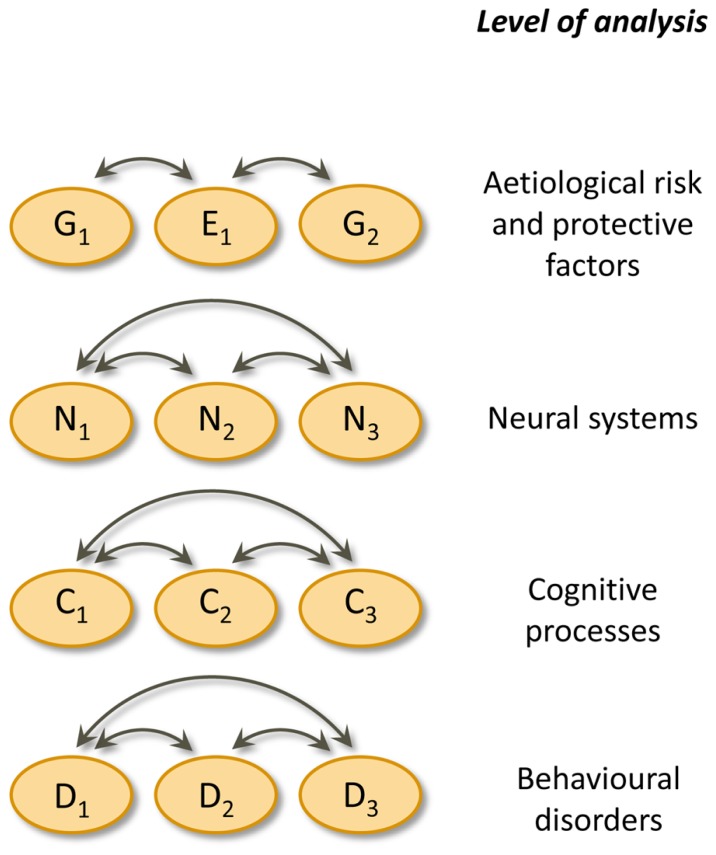
**Pennington’s multiple deficit model.** Double headed arrows indicate interactions. Causal connections between levels of analysis are omitted. G = genetic risk or protective factor, E = environmental risk or protective factor, N = neural system, C = cognitive process, D = complex behavioural disorder.

It seems that single deficit models are untenable and must give way to multiple cognitive deficit models for understanding developmental disorders. The multiple cognitive deficit model proposed by Pennington (2006) is depicted schematically in **Figure 1**. In his model, multiple genetic and environmental risk factors operate probabilistically by increasing the liability to a disorder; conversely, protective factors decrease the liability. These etiological factors produce the behavioural symptoms of developmental disorders by influencing the development of relevant neural systems and cognitive processes. Importantly, there is no single etiological or cognitive factor that is sufficient to cause a disorder. Instead, multiple cognitive deficits (each due to multiple etiological factors) need to be present to produce a disorder at the behavioural level. Some of the etiological and cognitive risk factors are shared with other disorders. As a result, comorbidity among developmental disorders is to be expected, rather than something that requires additional explanations. Finally, from Pennington’s the multiple deficit model (MDM) it follows that “the liability distribution for a given disease is often continuous and quantitative, rather than being discrete and categorical” (Pennington, 2006, p. 404). Therefore, the threshold between affected and unaffected is rather arbitrary.

Note that there are other models out there to explain the co-occurrence of neurodevelopmental disorders or the covariance of their associated continuous traits. For instance, Pennington et al. (2005) and Pennington (2006) set out several models differing not only on the dimension of a shared or distinct cognitive deficit, but also of a shared or distinct etiology. The severity model discussed in the first section is among them; others are pleiotropy, cognitive phenocopy, synergy, and assortment. Disconfirmatory data for each of the models is given, leading to the proposed MDM.

Pennington (2006) concludes his paper by remarking that – in contrast to single deficit models – it remains challenging to test the multiple cognitive deficit model. The model is much more complex than single deficit models, which are attractively parsimonious, but this complexity is needed to account for the observations at the different levels of analysis. The model is universally applicable to developmental disorders, but therefore remains abstract. It is not specified which etiological factors, neural systems, and cognitive processes interact to produce a given disorder.

## TESTING THE MDM

We argue here that a line of inquiry that can contribute to testing and specifying the MDM are family risk studies. In family risk studies, children are followed who are at risk of dyslexia by virtue of having an immediate dyslexic family member (usually a parent). Such studies have shown that 34–66% of them develop dyslexia (Scarborough, 1990; Elbro et al., 1998; Pennington and Lefly, 2001; Snowling et al., 2003; Torppa et al., 2010), depending on the stringency of the dyslexia criteria. The much higher prevalence of dyslexia among offspring of parents with dyslexia is consistent with twin studies showing moderate to strong heritability of dyslexia (e.g., Olson et al., 2014c).

From the MDM it follows that children at family risk experience at least some of the etiological risk factors: they inherit genetic risk factors and might experience a less rich literacy environment. Hence, it is hypothesized that at-risk children have a higher genetic and environmental liability than children without a family history of dyslexia (labeled control children). Furthermore, the at-risk children who go on to develop dyslexia are expected to show cognitive deficits (to varying degrees) in several processes. Some of these cognitive processes are expected to be affected even before the onset of reading instruction, as a consequence of etiological risk factors and deficient neural systems.

A key prediction of the MDM for family risk studies concerns the at-risk children who do not develop dyslexia. If liability to dyslexia were discrete (as would happen if only one factor, say a gene, were involved), at-risk non-dyslexic children would not differ from controls. However, according to the MDM, liability is continuously distributed. This also follows from the fact that reading ability is influenced by many genes of small effect, producing normal distributions of phenotypes (Plomin et al., 2008, p. 33). Consequently, the MDM predicts that at-risk children without dyslexia also inherit at least some disadvantageous gene variants from their dyslexic parents, giving them a higher liability than control children, although still lower than at-risk dyslexic children. At the behavioural and cognitive level this should translate into mild deficits in literacy skills and some of its cognitive underpinnings. When plotting mean performances of the three groups, a step-wise pattern (i.e., at-risk dyslexic < at-risk non-dyslexic < controls) would support a continuum of liability, one of the characteristics of the MDM.

Comparing the three groups of children on behavioural measures sheds light on cognitive deficiencies and behavioural symptoms, the bottom two levels in **Figure 1**. These three groups have also been compared on neural processing of visual and auditory stimuli (e.g., Regtvoort et al., 2006; Leppänen et al., 2010; Plakas et al., 2013), the second level of the MDM. Some family risk studies (e.g., Snowling et al., 2007; Torppa et al., 2007; van Bergen et al., 2011) have also examined aspects of the home environment, which belong to the etiological level. However, specific genetic risk factors remain hidden in family risk studies. As genetic screening of children for their dyslexia susceptibility is still far away, we propose an indicator of their genetic risk. Since reading ability is moderately to highly heritable and children receive their genetic material from their parents, we argue that cognitive abilities of parents can partly reveal their offspring’s liability. One, but maybe even both parents of at-risk children will have weaker reading skills than those of control children, reflecting selection criteria in family risk studies. However, the key issue is whether the reading skills of parents of at-risk children *with* dyslexia differ from the reading skills of parents of children *without* dyslexia. Based on the MDM it is expected that at-risk children who develop dyslexia have inherited more genetic risk variants than at-risk children without dyslexia and that this difference can be revealed by lower reading performance of parents of the at-risk dyslexic children. In Section “Parents’ literacy skills” we will elaborate upon parental effects.

Finally, the MDM predicts that some of the cognitive processes related to dyslexia are specific to dyslexia (or reading ability in general) and others are shared with comorbid neurodevelopmental disorders (and their accompanying continuous phenotype). In the following two paragraphs we will pursue the specificity matter, after which we return to the predictions for a family risk study laid out above.

## THE GENERALIST GENES HYPOTHESIS

One of the aims of dyslexia research is to identify cognitive processes playing a role in the developmental pathways that lead to dyslexia. The MDM states that some cognitive deficits are shared among disorders. This raises the question of *which* cognitive precursors of dyslexia are distinct and which are shared with other disorders. With regard to learning abilities, like reading ability, there is a hypothesis that addresses this specificity issue: the generalist genes hypothesis (Plomin and Kovas, 2005; Kovas and Plomin, 2007).

The generalist genes hypothesis states that the same set of genes is largely responsible for individual differences in learning abilities (i.e., pleiotropy). It stems from behavioural genetic studies employing the twin design. The twin design is the major method to quantify genetic and environmental influences on a trait. If for a certain trait MZ twins are more similar than DZ twins, genetic factors must play a role. If there is no difference in resemblance heritability is negligible. Estimates for the heritability of reading ability are in the range of 0.47–0.84 (Taylor et al., 2010; Byrne et al., 2009, respectively).

As a side note, it should be borne in mind that the high heritability of reading performance does not imply at all that educational improvements are pointless. Instead, they positively impact on almost all children’s reading achievement and raise the *average* of standardized scores of a class receiving effective reading intervention. Nonetheless, it is likely that individual *differences* among children remain largely genetically driven (Olson et al., 2009). This suggests that children with a genetic constraint on their reading development need increased reading instruction (as investigated by Zijlstra et al., under review).

Recently, the field of behavioural genetics has moved beyond quantifying genetic and environmental influences on a trait to studying genetic and environmental overlap between traits. For the three learning abilities reading, arithmetic, and language, empirical data have shown that the genes important for one learning ability largely overlap with the genes important for the other learning abilities. The genetic correlation is the measure that quantifies this: it indexes the extent to which genetic influences on one trait overlap with the genetic influences on another trait (independently of the heritability of the traits). The genetic correlation between learning abilities is about 0.70 (Plomin and Kovas, 2005; Kovas and Plomin, 2007). This suggests that roughly 70% of the genes associated with reading ability are generalists: they also influence other learning abilities. Hence Plomin and Kovas (2005) named their hypothesis the “generalist genes hypothesis.” As genetic correlations are not 1.0, there are also specialist genes: genes that contribute to dissociations among learning abilities.

Observed differences in learning abilities among individuals are also partly due to differences between the environments in which individuals were born, were brought up and live. Behavioural genetics subdivides environmental influences into those that make family members similar (called shared environmental effects) and those that do not contribute to resemblance among family members (called non-shared environmental effects). Also for these environmental components statistics exist analogous to genetic correlation. Shared environmental correlations among learning abilities are as high as genetic correlations, so shared environmental effects are also largely general effects (Kovas and Plomin, 2007). In contrast, non-shared environmental correlations are low. This indicates that these effects primarily act as specialists, contributing to performance differences in learning abilities within a child (Kovas and Plomin, 2007).

## THE HYBRID MODEL

**FIGURE 2:**
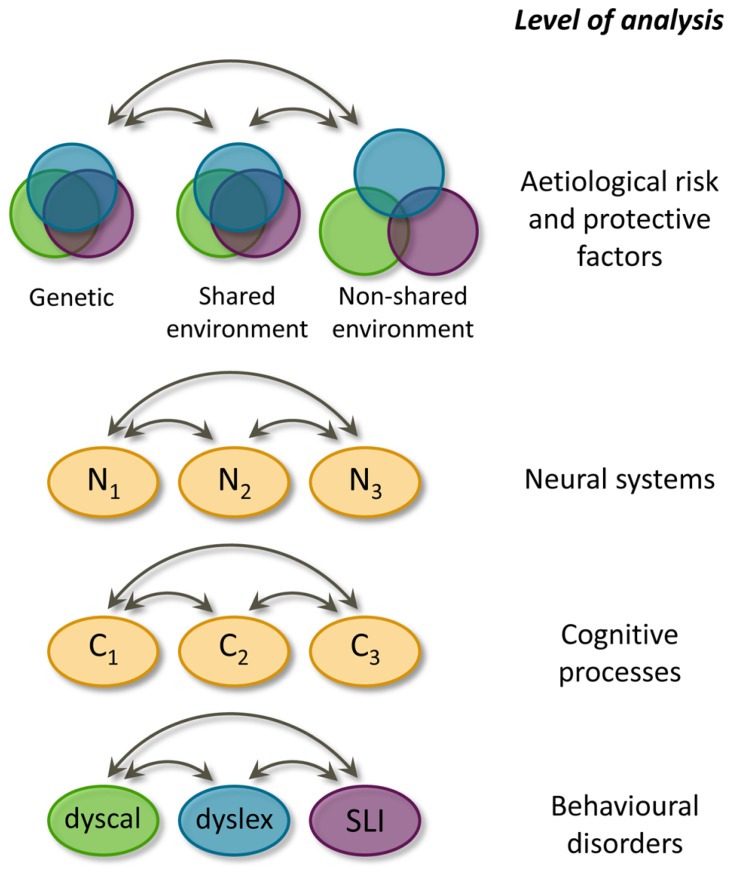
**Hybrid model for learning (dis)abilities, incorporating the generalist genes hypothesis (Kovas and Plomin, 2007) and the multiple deficit model (Pennington, 2006)**. Overlap at the aetiological level is graphically presented as Venn diagrams for each of the three sources of biometric variation. Double headed arrows indicate interactions. Causal connections between levels of analyses are omitted, N = neural system, C = cognitive process, dyscal = dyscalculia, dyslex = dyslexia, SLI = specific language impairment.

The generalist genes hypothesis and the multiple cognitive deficit model complement each other well. The MDM is more general because it holds for all common developmental disorders, while the generalist genes hypothesis specifically pertains to learning abilities and disabilities. Furthermore, the MDM includes four levels of explanation, whereas the generalist genes hypothesis only explicitly models the etiological level. Although the MDM also comprises polygenicity and pleiotropy, the generalist genes hypothesis *quantifies* for learning abilities the degree of overlapping and unique influences in each of the three etiological components (genetical, shared environmental, and non-shared environmental influences). We have visualized the generalist genes hypothesis and incorporated it into the MDM, yielding the hybrid model depicted in **Figure 2**. In this model only the first and the fourth layer are further specified because the generalist genes hypothesis only deals with these two levels. The etiological factors of the first level influence the behavioural manifestations at the fourth level by acting through the second and third level.

The hybrid model quantifies the overlap in etiological factors between learning abilities: genetic and shared environmental effects are largely shared by the three learning domains, whereas the non-shared environmental effects are largely distinct. These differential overlaps are visualized in the hybrid model as the degree of overlap between the circles. Despite this quantification of etiological overlap, the hybrid model does not specify, which etiological factors are relevant. Regarding genetic factors, molecular genetic studies will ultimately inform us which genes are implicated in dyslexia. Knowledge of specific genes contributing to dyslexia susceptibility promises to help bridge the gap from genes to neural systems, cognitive processes, and behavioural outcomes (Fisher and Francks, 2006).

Insight into which specific neural systems, cognitive skills, and behavioural symptoms are implicated in dyslexia can be gained from family risk studies. The hybrid model points to the opportunity to study reading in combination with arithmetic or language to increase insight into shared and distinct factors. We chose to focus on reading and arithmetic, both basic school skills are central during early primary school. As the model suggests, its disorders, dyslexia, and dyscalculia, indeed often co-occur (Landerl and Moll, 2010). Moreover, this pair of comorbidity is under-researched compared to the comorbidity of dyslexia with ADHD or language disorders. We aimed to study the comorbidity issue at the cognitive level of explanation. We investigated whether known precursors of reading are specific for reading or are shared between the development of reading and arithmetic.

## FINDINGS FROM A FAMILY-RISK STUDY

As argued above, a study with children with and without a family history of dyslexia is valuable in relation to the MDM (or hybrid model), because specific testable hypotheses follow from the model. To reiterate, the following four hypotheses followed from Section “Testing the MDM”:

1. Group comparisons on children’s reading and reading related skills show a step-wise pattern (i.e., at-risk dyslexic < at-risk non-dyslexic < controls).
2. At-risk children with dyslexia exhibit more than one deficit.
3. Some deficits are reading specific and others are shared with e.g. arithmetic (dis)ability.
4. Group comparisons on parents’ reading and reading related skills show a step-wise pattern.

The first three hypotheses pertain to the children, whereas the fourth hypothesis concerns the parents.

As an illustrative example we will present a family risk study that speaks to all four hypotheses. The family risk study is part of the Dutch Dyslexia Programme, abbreviated DDP (for an overview, see van der Leij et al., 2013). The study employs a prospective design, in which the progress of children (*N* = 212) at high and low family risk is followed. Children were considered at high family risk if (at least) one of their parents and another family member had dyslexia. After two and subsequently 3 years of reading instruction they were categorized as either dyslexic or non-dyslexic (below or above the 10th percentile cut-off on word-reading fluency, respectively). Subsequently, they were compared concurrently and retrospectively with each other and with typically developing children without such a family background. In the present paper, we will focus on the findings regarding reading and reading related (cognitive precursors and correlates of dyslexia) in parents and children (van Bergen et al., 2012, van Bergen et al., 2014a,b). We investigated the cognitive profile characteristic of the three groups of children and the impact of the cognitive profile of parents and the literacy environment parents create on children’s reading outcome. An overview of the findings is given in **Table 1**.

**Table 1:** Overview of the behavioural and cognitive findings from the DDP family risk study.

			Findings				
			Longitudinal predictor of reading^a^		Longitudinal predictor of arithmetic^a^	
Who	When	Variable	On its own	After controlling for arithmetic	On its own	After controlling for reading	Group differences^b^	Support for MDM?
Child	Age 4 - not yet in school	Nonverbal IQ^c^	✓	✗	✓	✗	FRD < FRND = C	✗
		Verbal IQ^c^	✓	✓	✓	✗	FRD < FRND < C	✓
	End kindergarten (age 5–6)	Rapid naming^d^	✓	✓	✓	✓	FRD < FRND = C	✗
		Phonological awareness^d^	✓	✗	✓	✗	FRD < FRND < C	✓
		Letter knowledge^d^	✓	✓	✓	✗	FRD < FRND = C	✗
	End Grade 2 (age 8)	Rapid naming^e^	–	–	–	–	FRD < FRND = C	✗
		Phonological awareness^e^	–	–	–	–	FRD < FRND < C	✓
		Word-reading accuracy^e^	–	–	–	–	FRD < FRND < C	✓
		Word-reading fluency^e^	–	–	–	–	FRD < FRND < C	✓
		Pseudoword-reading fluency^e^	–	–	–	–	FRD < FRND < C	✓
		Spelling^e^	–	–	–	–	FRD < FRND < C	✓
Dyslexic parent		Rapid naming^e^	–	–	–	–	FRD < FRND (< C)	✓
		Nonword repetition^e^	–	–	–	–	FRD = FRND (< C)	✗
		Word-reading fluency^e^	–	–	–	–	FRD < FRND (< C)	✓
		Pseudoword-reading fluency^e^	–	–	–	–	FRD = FRND (< C)	✗
		Spelling^e^	–	–	–	–	FRD = FRND (< C)	✗
		Self-reported literacy difficulties^d^	–	–	–	–	FRD = FRND (< C)	✗
Non-dyslexic parent		Self-reported literacy difficulties^d^	–	–	–	–	FRD < FRND (= C)	✓

a Reading = word-reading fluency, arithmetic = arithmetic fluency.

b Dyslexia status was assessed using word-reading fluency at the end of Grade 2 or halfway Grade 3. Regarding the parents of the control children, the best-reading parent features in the non-dyslexic parent comparison, and the other in the dyslexic-parent comparisons.

c Reported in van Bergen et al. (2014b).

d Reported in van Bergen et al. (2014a).

e Reported in van Bergen et al. (2012).

*FRD = familial-risk dyslexia, FRND = familial-risk no-dyslexia, C = control, MDM = multiple deficit model.*

### CHILDREN’S (PRE)LITERACY SKILLS

The MDM predicts that normal reading children with a family risk do slightly poorer on reading and spelling than normal reading children without such risk. In addition, they are assumed to perform more poorly on reading related skills as there is evidence that these underlying cognitive processes of reading are also complex traits, influenced by multiple genetic and environmental factors (Petrill et al., 2006; Naples et al., 2009). Whether such a step-wise pattern was observed is indicated in the last two columns of **Table 1**.

At the end of Grade 2, the at-risk children with dyslexia were severely impaired compared to control children on all measures of accuracy and fluency of (pseudo)word reading (van Bergen et al., 2012; **Table 1**). In addition, they made many errors in spelling words. Although the at-risk group without dyslexia had literacy skills within the normal range for their age they read significantly less accurately and fluently than controls on all of these reading measures. The same step pattern was found for spelling. Thus, the MDM-based hypothesis about the at-risk no-dyslexia group taking up an intermediate position between the other two was confirmed.

Importantly, we also found a stepwise pattern in the frequency of the comorbid disorder of dyscalculia (van Bergen et al., 2014ca). Of the dyslexic children, 42% of the children performed below the 10th percentile on a calculation fluency test. In the FR-nondyslexic group this was 20%, which was significantly above the 8% in the group of control children. Such a stepwise pattern is to expected as, according to the MDM, comorbidity is due to shared risk factors of both disorders and, consequently, a familiar risk for one disorder also leads to an elevated risk for the other disorder.

With regard to the reading related skills, we included the most important precursors and correlates of dyslexia: phonological awareness (i.e., the blending and segmentation of speech sounds), rapid naming of familiar items (i.e., colors and digits) and letter knowledge. Letter knowledge was assessed at the end of kindergarten (age 5 or 6), before the start of reading instruction. The at-risk dyslexic group lagged behind on letter knowledge, whereas the at-risk children without later dyslexia showed a normal level of knowledge. The absence of a stepwise pattern is not in accordance with the MDM model. However, it could be argued that letter knowledge should be regarded as belonging to the symptom level, being a forerunner or autoregressor of reading.

Phonological awareness and rapid naming were assessed at the end of kindergarten (age 5 or 6) and at the end of Grade 2. On both occasions the findings were similar. The at-risk children without later dyslexia showed normal rapid naming, but performed below controls on phonological awareness. The at-risk dyslexic group was impaired on both skills as compared to the other two groups. Note that because the cognitive deficiencies in the dyslexic group were already in place in kindergarten, before the start of reading instruction, they are due to etiological factors rather than being the consequence of poor reading and less print exposure.

Apparently, phonological awareness is associated with both reading and risk status, while rapid naming is only related to reading status. The fact that rapid naming does not fit the MDM prediction in the DDP and in the family risk study of Moll et al. (2013) calls for an explanation. One possibility is that the at-risk children who go on to develop normal reading skills might do well despite their family risk because the efficiency of the processes that rapid naming tap might protect them against dysfluent reading. Their mild literacy problems could be due to their mild phonological awareness deficit. Another possibility is that, in contrast to the protective explanation, rapid naming is not a protective or risk factor, nor causally implicated, but an integral part of the reading system (see Section “The Intergenerational Multiple Deficit Model” for more on the reading system). On this view, Norton and Wolf (2012) conceptualize rapid naming as “a microcosm or mini-circuit of the later-developing reading circuitry” (p. 430).

We also examined the relation between more general abilities, verbal and nonverbal IQ, around the age of four, and reading outcome at the end of Grade 2 (see van Bergen et al., 2014cb). It was found that at-risk children who go on to become dyslexic were impaired relative to controls on both verbal and nonverbal IQ, with the gap being larger for verbal IQ. The at-risk children who do not become dyslexic showed good nonverbal abilities, but their verbal IQ was slightly but significantly lower than that of controls. For a discussion about the nature of the link between early IQ and subsequent reading the interested reader is referred to van Bergen et al. (2014b).

In the MDM comorbidity is explained by shared risk factors. To pursue this issue, it was examined whether children’s skills before the onset of reading instruction were specifically related to reading. It appeared that nonverbal IQ was equally strongly related to later reading achievement (e.g., word-reading fluency) as to later arithmetic achievement (e.g., arithmetic fluency), while verbal IQ was specifically predictive of reading. With respect to the preliteracy skills, all were shown to be predictive of later arithmetic achievement as well. Rapid naming was equally strongly related to reading and arithmetic, but phonological awareness and letter knowledge were more specific precursors of reading (van Bergen et al. (2014a). Thus, some of the cognitive processes of importance to reading are also important for arithmetic, whereas others are distinct to reading. This is in line with the MDM (Pennington, 2006), the generalist genes hypothesis (Plomin and Kovas, 2005; Kovas and Plomin, 2007) and hence also with the hybrid model (**Figure 2**). Nonverbal IQ and rapid naming are shared and therefore contribute to the correlation between arithmetic and reading. Likewise, at the lower end of the distribution, they contribute to the comorbidity between dyscalculia and dyslexia. Verbal IQ, phonological awareness, and letter knowledge were found to be skill-specific cognitive processes, contributing to the dissociation between arithmetic and reading. Rapid naming is an interesting case, as part of what it taps is shared between reading and arithmetic, but it also measures processes specific for each of the two academic domains (see **Table 1**).

### FAMILY CHARACTERISTICS

In addition to predictors of dyslexia residing in children we examined possible predictors in their families. More specifically, we studied effects of home literacy environment and parental literacy skills on children’s reading outcome.

#### Home literacy environment

In short, the three groups did not differ on cognitive stimulation by parents, but there was a tendency for parents of control children to own more magazines, newspapers and books. The two at-risk groups did not differ in any of the measures of home literacy environment (van Bergen et al., 2014a). Our findings are in agreement with findings from other family risk studies, which also failed to show effects of home literacy environment on children’s reading outcome (Elbro et al., 1998; Snowling et al., 2007; Torppa et al., 2007; van Bergen et al., 2011). Thus, no environmental risk factors of substantial effect have been identified that would have been easy targets for intervention. Although behavioural genetic studies point to substantial heritability of reading, they also estimate that roughly 30% of individual differences is due to environmental factors (Petrill et al., 2006; Taylor et al., 2010; Olson et al., 2014c). The moderate total environmental influence and small to negligible shared-environmental influence do not leave much room to find effects of home literacy environment. Also other environmental factors warrant further investigation, such as pre- and perinatal factors and school and classroom characteristics.

#### Parents’ literacy skills

The key innovating factor of the DDP family risk study is probably the inclusion of cognitive abilities of the parents. We went beyond using parental literacy for the sole purpose of dichotomizing children into high and low family risk samples by examining the relation between reading and reading-related skills of the parents and reading skills of their children. We had objective measures of the parents with dyslexia. Although all children in the at-risk sample have a parent with dyslexia, they might still vary in their degree of family risk for dyslexia. We tested this by comparing the groups of at-risk children with and without dyslexia on the reading skills of their parent with dyslexia. Since parents pass on their genes to their offspring and shape their environment, parental reading skills might be taken as an indicator of the offspring’s liability to dyslexia.

In a previous family risk study (van Bergen et al., 2011) the dyslexia of the parents of the affected children was more severe than the dyslexia of the parents of the unaffected children, yielding the stepwise pattern predicted by MDM. This is a striking finding, because the affected parents read on average at the fifth percentile compared to national norms. Yet even in this restricted range group differences were observable.

In the DDP sample the difference between the at-risk children with and without dyslexia was replicated for the affected parent’s word-reading fluency (see van Bergen et al., 2012). The two at-risk groups did not differ in parental pseudoword reading. They did not differ in spelling, and non-word repetition either, though both groups were impaired compared to controls. Interestingly, however, the parents of the at-risk dyslexia children were slower on rapid naming than those of the at-risk no-dyslexia children. This underscores the special role of rapid naming, at least in transparent orthographies.

In the two above mentioned studies data were reported of the parent with dyslexia. The study of van Bergen et al. (2014a) completes this by examining the influence of the parent *without* dyslexia for the first time. As hypothesized, also for the non-dyslexic parents there was a difference between the two at-risk groups: the parents of the affected children reported more literacy difficulties compared to those of the unaffected children.

The results concerning the unaffected parent further support the conclusion that children at family risk for dyslexia differ in their liability, as indicated by differences in parental reading skills between at-risk children with and without dyslexia. Moreover, differences between the two family risk groups in the severity of the dyslexia of the affected parent have now been replicated in Finnish. Torppa et al. (2011) showed differences in parental reading fluency, accuracy, and spelling.

Do the findings regarding precursors in families lend support for the MDM? According to this model, the etiology of dyslexia (and other developmental disorders) is multifactorial and probabilistic. Multiple genetic risk variants interact with each other and with multiple environmental risks to ultimately produce the disorder at the behavioural level. Some environmental factors were measured directly but did not have an effect. Genetic risk factors were not measured directly. Although there is now a huge body of evidence indicating that genes contribute importantly to individual differences in reading ability (Hayiou-Thomas et al., 2010; Olson et al., 2014c), the specific gene variants found thus far only explain a tiny part of these differences (see Bishop, 2009, for the example of the *KIAA0319* gene), despite substantive work in the field of molecular genetics (for a recent overview, see Carrion-Castillo et al., 2013). This phenomenon also applies to other common traits and is called the mystery of the missing heritability (see e.g., Manolio et al., 2009). Genetic screening is therefore not (yet) informative about a child’s genetic vulnerability to dyslexia (Bishop, 2009). Instead, we propose that since parents pass on their genetic material to their offspring and shape their environment, cognitive abilities of parents could be used as an overall indicator of the genetic and environmental risk and protective factors in the MDM.

The DDP study provides two kinds of support for parental skills being an indicator for children’s liability. First, as in other family risk studies, two samples of children were recruited based on having or not having a parent with dyslexia. The current and previous family risk studies (Scarborough, 1990; Elbro et al., 1998; Pennington and Lefly, 2001; Snowling et al., 2003; Torppa et al., 2010) found a large effect of having a family history on children’s risk of becoming dyslexic. For example, in the DDP study it was found that the rate of dyslexia was 30% in the high-risk group and only 3% in the low-risk groups (van Bergen et al., 2012). Thus, having a parent with dyslexia increases the risk considerably. Secondly, within the at-risk sample it was found that affected *and* unaffected parents of the affected children had more literacy problems than those of the unaffected children. Moreover, when considering at-risk children’s reading fluency on a continuous scale (rather than having or not having dyslexia), parental reading skills were significant predictors of children’s reading skills.

Our findings thus support the view that skills of parents indicate their offspring’s liability, which in itself is the combination of all genetic and environmental factors that affect reading development. Therefore, parental skills might shed light on the etiological level in the MDM. But based on parental skills it is not possible to disentangle the genetic and environmental contribution to the intergenerational transmission of skills. However, according to our data the transmission of risk seems to be mainly via genes, including gene-environment correlation (see also Figure 1 in Lyytinen et al., 1998). It is important to note that genes are inherited, not phenotypic traits. Thus, although the DDP data reinforce the view that parental skills are indicative of their offspring’s liability, parental skills will never completely specify it.

## THE INTERGENERATIONAL MULTIPLE DEFICIT MODEL

In our opinion there are two omissions in the MDM (Pennington, 2006) when applied to dyslexia. The first one – only touched upon here – relates to the reading system and the second to intergenerational transfer, which will be discussed in the remainder of this article.

First, when modeling reading ability and disability, the boxes at the level of cognitive processes in the MDM are typically thought of as precursors or correlates of reading, such as phonological awareness and rapid naming (e.g., Willcutt et al., 2010), or the cognitive components that each of these tasks tap. How these tasks and their components relate to reading outcome is extensively studied. In parallel, there is an extensive body of research into computational models of the reading system, in which visual word recognition is simulated (e.g., Coltheart et al., 2001; Peterson et al., 2013; Ziegler et al., 2014). Computational models are evaluated by how well they predict experimentally observed characteristics of the reading system, like lexicality and word length effects. The reading process is an important link between underlying cognitive process (such as phonological awareness and rapid naming) and the outcome of the reading process, reading accuracy and speed. Hence, in a MDM of reading (dis)ability, the cognitive level could be split up into underlying cognitive processes and the reading system (see Jackson and Coltheart, 2001). Research into underlying cognitive processes and into the reading system have developed separately, but van den Boer et al. (2013) recently made an important first step in linking these fields.

**FIGURE 3:**
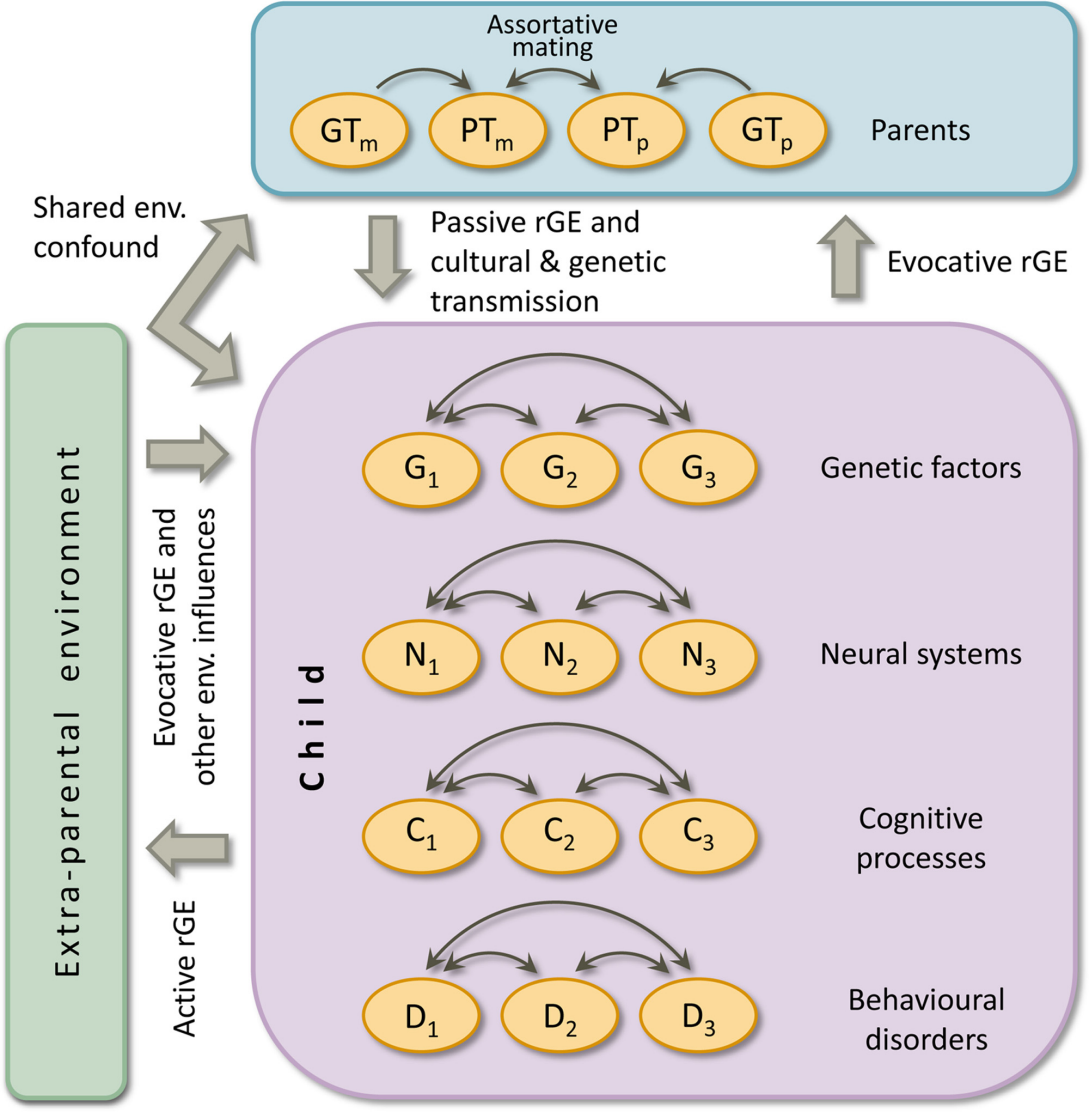
**The intergenerational multiple deficit model.** Double headed arrows indicate interactions. Causal connections between levels of analyses are omitted. GT_m_ = maternal genotype, PT_m_ = maternal phenotype, GT_p_ = paternal genotype, PT_p_ = paternal phenotype, G = genetic risk or protective factor, N = neural system, C = cognitive process, D = complex behavioural disorder, env. = environmental, rGE = gene-environment correlation. Terminology: a *phenotype* is any measurable characteristic of an individual (e.g., reading ability or parenting style); a *genotype* is an individual’s genetic makeup. There is *shared environmental confound* if an environmental factor influences both the parental and child phenotype. *Genetic transmission* refers to the genotypic factors passed down from parent to offspring that influence the phenotypes in both generations. *Cultural transmission* is the genuine environmental influence of parental characteristics on child outcome, so controlled for environmental and genetic confounds. *Assortative mating* is non-random mating. *Gene-environment correlation* (rGE) refers to the situation in which exposure to environments is not independent but correlated to the child’s genotype (see the text for explanation about the three forms of rGE). The figure depicts the situation for one individual child and his/her (biological) parents. At the group level (i.e., multiple children), a second form of gene-environment interplay emerges: *gene-environment interaction*. That is, heredity depends on the environment, or sensitivity to the environment depend on genotype.

Secondly and applicable to all complex developmental disorders, intergenerational transmission of risk and protective factors is not explicitly present in the MDM, as it focusses on an individual child. Therefore, we propose an extension of the MDM: the intergenerational MDM (iMDM). Below we will elaborate on this model that is depicted in **Figure 3**. In the figure it can be seen that a top layer is added to Pennington’s MDM, which represents characteristics of parents. The environment as created by parents is included in the top layer; other environmental factors are placed on the side. Note that again influences between child layers are omitted from the figure.

Cognitive abilities of parents, for instance reading ability, form part of their phenotype (PT in **Figure 3**). Their phenotype is the result of their genotype (GT) in interaction with their environment. Genes do not code for cognitive and behavioural traits but for the structure of proteins and the regulation of gene expression, which in highly complex ways and in interaction with the environment guides the building and maintenance of the brain (Fisher, 2006; Fisher and Francks, 2006). Despite this gap between genes and cognition, for traits that show genetic influences in behavioural genetic studies there must be a relationship between genotypic and phenotypic variation. In other words, for heritable traits parental phenotype is a proxy for their genotype. As both parents pass on half of their genes to their offspring, the genotype of both of them determines the genotype of their offspring. It follows that the phenotype of parents must be related to some extent to the genotype of children, which includes children’s genetic risk and protective factors for a particular developmental disorder.

In addition to transmission of parental skills via genetic pathways, parental skills could be passed on via environmental pathways. Parents largely shape their children’s childhood environment, which creates a relation between parents’ characteristics and children’s environment. This environment could exert a direct environmental effect (i.e., genetically unconfounded), referred to as *cultural transmission*. Hence, the cognitive phenotype of parents could be one of the factors that determines children’s environmental risk and protective factors. The environment created by parents could also be correlated with the genotype of both parents and offspring, creating what is called (from the offspring’s perspective) a *passive gene-environment correlation*. For example, good reading parents are more likely to spend a lot of time reading, thereby providing a role model to their children. Moreover, they appear to be better educated, and as a result, might live in better neighborhoods and might send their children to higher achieving schools. The family environment a child is exposed to can also be correlated with both generation’s genotypes by parental behavior elicited by the child. Sticking with our reading-ability example, children genetically inclined to become fluent readers may be more likely to ask to be read to early on and ask for books and library visits later on. This phenomenon is termed *evocative gene-environment correlation.* Other aspects of the phenotype of parents might also be associated with or directly influence children’s reading development. For instance, the behavior of parents and the interaction between them determines how structured or chaotic the household is, a factor that has been shown to be related to children’s school performance (Hanscombe et al., 2011). Apart from genetic and cultural transmission, a third contributor to parent-child resemblance is shared *environmental confound* (D’Onofrio et al., 2003). In the case of reading, poverty could limit access to printed and digital reading material, which could affect reading ability in both generations. In conclusion, the phenotype of parents must also be related to a certain degree to children’s environmental risk and protective factors. In addition, the mechanisms discussed highlight that measures of the environment that relate to child outcome may be attributable to familial confounding, rather than causation.

Given the above two lines of reasoning, the phenotype of parents is informative about children’s genetic and environmental factors. Focussing on developmental disorders, this suggests that certain aspects of the phenotype of parents can inform us about a child’s liability to a particular developmental disorder. Regarding dyslexia, the phenotypic aspects of parents that are expected to shed light on children’s liability to dyslexia are skills in accurate and fluent reading, spelling, and their cognitive underpinnings like phonological awareness and rapid naming. Related skills (such as language and arithmetic) and their underlying cognitive abilities might also play a role. The ability of parents on each of the relevant continua can be conceptualized as a position in multivariate space. The position of father and mother in multivariate space is proposed to be indicative of a child’s predisposition towards dyslexia.

Apart from environmental exposure closely linked to parental characteristics, children experience other environmental factors that influence their development. In **Figure 3** these extra-parental influences are put in the box on the side. They can influence all four child levels. In the case of reading, one can think of quality of the school and teacher, reading-instruction method, access to print and digital media, and factors related to child development in general, like (other) caretakers, peer influences, accidents, nutrition, and toxic threats.

Infants’ environment is almost exclusively shaped by their parents, but as children grow older their environment becomes increasingly shaped independent by their parents. First, by gaining independence, running from acquiring locomotion to living independently. Second and related, by spending more and more time away from parents. This illustrates that children more and more actively select and create their environment. If this environmental exposure is correlated with the child’s genotype, this is called *active gene-environment correlation*. For instance, children with a high genetic potential for good reading may actively seek out for opportunities to read. Children’s genetically influenced abilities may also elicit environmental responses from others than their parents. For example, good readers may be given more difficult reading material by teachers, a form of *evocative gene-environment correlation*.

A form of gene-environment interplay not discussed so far is *gene-environment interaction*. This refers to a moderator phenomenon in which sensitivity to an environment depends on one’s genotype (e.g., resilience to poor education), or the corollary, heritability depends on environmental exposure. An example of the latter in dyslexia research is a study by Friend et al. (2008) who found higher heritability of dyslexia among children from high compared to low socio-economic status. Gene-environment interaction is a group-level phenomenon and is therefore not depicted in the iMDM (**Figure 3**), which displays processes within the triad of an individual child and his/her (biological) parents, as well as the child-specific environment. As an illustration, the findings of Friend et al. would translate in an iMDM with strong genetic transmission for dyslexia predisposition in a child from a high socio-economic status family.

The iMDM is inspired by the described DDP study on dyslexia, but is generally applicable to other multifactorial developmental disorders with a genetic component. Examples of such disorders include ADHD, developmental coordination disorder, dyscalculia, SLI, and autism spectrum disorder. With respect to autism spectrum disorder, a number of studies (e.g., Happé et al., 2001; Bölte and Poustka, 2006; Losh et al., 2009) have studied the cognitive phenotype of parents of probands (as opposed to children of probands, as in family risk studies of dyslexia) and found in parents similar but milder impairments as in their children, indicating parent-child resemblance. A second example concerns SLI. Bishop’s group found that language skills of probands and their parents were correlated (Bishop et al., 2012) and that a parent’s non-word repetition ability was a predictor of whether the child would develop SLI (Bishop et al., 2012). These examples provide evidence of intergenerational transfer of cognitive skills other than reading.

The advantage of generally applicable MDMs comes however at a cost. First, the model is still empty and has to be specified for each particular (set of) developmental disorder(s). Candidate ingredients for the case of dyslexia are given throughout the current paper for the cognitive level. For the genetic level, the reader is referred to Carrion-Castillo et al. (2013), for the neural-system level to Richlan (2012), and for bridging these levels to Giraud and Ramus (2013). Second, the model (as depicted in **Figure 3**) is difficult to prove wrong. Still, the iMDM can inform the building of structural equation models for family data. Competing models can be tested to see which model best fits the observed data. Importantly, for (a) specific developmental disorder(s) the iMDM can therefore be falsified.

## FUTURE AVENUES OF RESEARCH

Despite that Pennington’s MDM as such is difficult to falsify, it has initiated a large body of research and our hope is that the intergenerational extension will further fuel this movement. Pennington’s model has stimulated research in which more than one level of analysis is incorporated (vertical expansion) and has especially boosted horizontal expansion of studies, investigating more than one disorder simultaneously to understand comorbidity. By doing so, one can uncover shared and distinct risk factors at each of the levels of explanation. This not only helps to understand the origin of the comorbidity, but also the developmental paths leading to each of the disorders. For instance, a developmental disorder could develop secondary as a result of a primary disorder, or the two co-occur because of shared etiological factors (as evidenced by genetic correlations and environmental correlations). In examining the specificity of precursors for dyslexia we included arithmetic and dyscalculia in the DDP, but comorbidities with other developmental disorders were not investigated. Including more than just a single (dis)ability in future work will enhance our understanding.

From a practical point of view, the iMDM sheds light on an additional way to estimate disorder risk: not only an individual child’s precursors to a certain disorder carry predictive power to identify young children at risk, also the cognitive and behavioural profile of their parents indicate risk. Studying intergenerational transfer and comorbidity can also be combined: parents might confer risks in different cognitive domains, like reading, language, and attention and additionally, parenting practices might not be optimal. Therefore, future studies are needed to test whether a more complete picture of parents’ cognitive and behavioural profile yields a more reliable indication of their offspring’s liability to a particular disorder. Reliable assessment of liability is of clinical importance: young children identified as at high risk can be enrolled in an intervention programe to ameliorate the risk. In the case of dyslexia, Zijlstra et al. (under review) showed in high risk children that trying to prevent reading difficulties works better than remediating once children lag behind substantially.

Apart from inspiring clinical work, the iMDM draws attention to an interesting area for future fundamental research: investigating transmission from one generation to the next. Research incorporating phenotypic characteristics of parents alongside one or more analysis levels in offspring is still sparse. We have discussed some family studies that revealed traits that show intergenerational correlations. Future family studies can further explore familial transmission patterns to identify relevant parental phenotypes and quantify phenotypic intergenerational associations. If both parents are assessed, one can test firstly whether this intergenerational association is moderated by the gender of parent and/or child. For example, van Bergen et al. (2014c) recently reported that paternal reading ability (as indicator for offspring’s genetic liability) was a better predictor of offspring’s reading ability for higher-educated fathers. Interestingly, this interaction was absent for mothers. The observed interaction for fathers is line with the gene-environment interaction between socio-economic status and heritability of dyslexia found by Friend et al. (2008). The differential pattern for fathers and mothers demonstrates that parental influences can be parent specific.

Secondly, if data on both parents are collected one can test whether there is *assortative mating* for the trait under study (see the correlation in **Figure 3** between maternal and paternal phenotypes). For level of education for example, the intuitive idea that people tend to choose a partner with similar academic attainment has been confirmed (e.g., Mare, 1991). Regardless of the iMDM it is important to establish the degree of assortative mating because it biases heritability estimates if not accounted for (Plomin et al., 2008, p. 160).

Genetically informed family studies can ultimately disentangle the contributions of causal genetic and environmental effects and gene-environment correlations to such an intergenerational correlation. Two examples of genetically sensitive family studies that rigorously investigate the mechanisms responsible for parent-offspring resemblance are studies which include MZ and DZ twin children plus their parents (nuclear twin-family design), or studies with adult MZ and DZ twins plus their offspring (children-of-twins design)^6^. Combining two such samples even allows for estimating cultural transmission and passive and evocative gene-environment correlation (Narusyte et al., 2008). That is, the direct environmental effect of parenting (or another parental trait) on children’s outcome can be estimated while controlling for familial confounds. Regarding gene-environment correlation, the direction of effect (see **Figure 3**) can be revealed. We are unaware of such studies in the field of learning (dis)abilities, but see for an example on parental depression and offspring psychopathology Silberg et al. (2010).

The next step in a genetically sensitive family design would be to test whether parents differ in the relative quantity of cultural and genetic transmission. To start with cultural transmission, it may be expected that the parent who has the largest share in the child’s upbringing exerts larger environmental influence. If parental involvement information is available, the structural equation models estimating cultural and genetic transfer could be rerun with parent couples subdivided based on involvement (rather than gender), or parental involvement could be included as a moderator. The amount of cultural transmission could also depend on the quality of the parent-child relationship and the gender of parent and child.

For genetic transmission, quantitative differences in transmission of paternal and maternal risk could arise from two mechanisms. First, if susceptibility genes show parent-of-origin effects [e.g., genomic imprinting, in which genes from mother and father have differential expression levels (Lawson et al., 2013)]. And second, if susceptibility genes would be carried on sex chromosomes (X and Y). Genetically informed family studies can estimate the total genetic risk that is passed down per parent. Hence, differences in transmitted genetic risk can be tested. Molecular genetic studies are needed to investigate the biological basis of possible maternal and paternal differences. Concerning dyslexia, the well-replicated candidate genes all lie on autosomal chromosomes, although there is some evidence for a locus on the X chromosome being implicated (Carrion-Castillo et al., 2013). Parent-of-origin effects have not yet been studied in relation to dyslexia (but see for a recent example on SLI Nudel et al., 2014).

To conclude, the iMDM encourages the inclusion of parent characteristics in future studies, which will enhance our prediction of risk and understanding of common neurodevelopmental disorders. The next exciting step is to conduct genetically informative family studies, in which genetic and environmental causal effects can be separated from familial confounding. This will bring us closer to elucidating causal chains underpinning disorder etiology.

## Conflict of Interest Statement

The authors declare that the research was conducted in the absence of any commercial or financial relationships that could be construed as a potential conflict of interest.

## Footnotes

6 The logic and modeling behind these approaches are beyond the scope of this article, but the interested reader is referred to D’Onofrio et al. (2003) for the children-of-twins design and D’Onofrio et al. (2013) for an overview of genetically informed family studies.
